# Physiology of Preaching

**Published:** 1871-08

**Authors:** 


### PHYSIOLOGY OF PREACHING.

Husband all your strength for the delivery of the sermon. Take not a step, sing not a line, speak not a word, unnecessarily; for we rise in the morning with a certain amount of physical power, and acquire but little more during the day from other sources. It is the recuperation which sleep gives, and if that force is expended in a long walk or ride to church, or in any muscular effort whatever, you have just that much less for the sermon; and every clergyman must know that the more physical vigor he has, the easier he can preach.

Singing is an effort, hence every verse sung is a loss of vocal power.

Be more of a man than to be the slave of a sip of water, a lozenge, or a lump of sugar, before or during preaching; the necessity of these grow upon a man with great rapidity, and detract from his independence and self-reliance.

Avoid conversation on any subject from the time of rising in the morning until the sermon is delivered; for the more your subject absorbs your whole being, the greater will be the unction with which the message is delivered.

Begin in a low tone, but with the utmost distinctness of utterance, and as the lungs grow warm and the vocal organs more pliable, throw in more voice gradually until the end of the discourse, otherwise you will break down before you are half done.

Never study a gesture or an intonation; this involves a mental diversion from the subject, and impairs your force.

If the closing prayer is a succinct reproduction of the great practical truth of the discourse, and that only, the chances are greatly increased of its long remembrance.

There are few pulpits which are free from draughts or eddies of air; preaching involves an increased perspiration of the body, and the danger is great, the injury certain, of cooling off too quickly, even before leaving the pulpit; hence both in winter and summer it is of incalculable advantage to have a thick cloak at hand, to be thrown over the shoulders the moment the seat is taken after the sermon has been delivered; perhaps it is of more importance in summer than in winter, because perspiration is so much more readily induced and is more apt to become profuse; hence the danger of cooling off is greatly increased.

Put on overcoat, hat, gloves, everything, before passing out of the door, and, if possible, walk home, walk briskly, mouth shut, breast protected.

It is suicidal to ride even a mile within an hour after preaching, if it is a chilly day or a cool wind is blowing.

A meal should be taken as soon after preaching as possible, if there has to be another sermon on the same day a mile or more distant.

If another sermon is to be preached in the afternoon at the same place, make your dinner of a cup or two of hot drink, a piece of cold bread and butter, and a slice of meat, nothing else whatever, under any pretense whatever; because in part the great flow of nervous power is towards the brain, and is kept up by the mind running back on the sermon; or it is directed with all the power left in the consideration of the sermon to be delivered, with the result that so little goes to the stomach that it is barely sufficient to digest a comparatively small meal, and that a very light one; if a hearty dinner is taken before an afternoon discourse, it remains for that reason undigested, decomposition of the food takes place, wind is evolved, distending the stomach, which presses up against the more yielding lungs, curtails their power of action, and there is such an uncomfortable sense of oppression as to unfit for the second service.

After the last service of the day, do all that is possible to get the mind out of the day's rut, by thinking of anything else than the labors of the day.

